# Defining Immunological Impact and Therapeutic Benefit of Mild Heating in a Murine Model of Arthritis

**DOI:** 10.1371/journal.pone.0120327

**Published:** 2015-03-20

**Authors:** Chen-Ting Lee, Kathleen M. Kokolus, Nicholas D. Leigh, Maegan Capitano, Bonnie L. Hylander, Elizabeth A. Repasky

**Affiliations:** Department of Immunology, Roswell Park Cancer Institute, Buffalo, New York, United States of America; Queen Mary University of London, UNITED KINGDOM

## Abstract

Traditional treatments, including a variety of thermal therapies have been known since ancient times to provide relief from rheumatoid arthritis (RA) symptoms. However, a general absence of information on how heating affects molecular or immunological targets relevant to RA has limited heat treatment (HT) to the category of treatments known as “alternative therapies”. In this study, we evaluated the effectiveness of mild HT in a collagen-induced arthritis (CIA) model which has been used in many previous studies to evaluate newer pharmacological approaches for the treatment of RA, and tested whether inflammatory immune activity was altered. We also compared the effect of HT to methotrexate, a well characterized pharmacological treatment for RA. CIA mice were treated with either a single HT for several hours or daily 30 minute HT. Disease progression and macrophage infiltration were evaluated. We found that both HT regimens significantly reduced arthritis disease severity and macrophage infiltration into inflamed joints. Surprisingly, HT was as efficient as methotrexate in controlling disease progression. At the molecular level, HT suppressed TNF-α while increasing production of IL-10. We also observed an induction of HSP70 and a reduction in both NF-κB and HIF-1α in inflamed tissues. Additionally, using activated macrophages *in vitro*, we found that HT reduced production of pro-inflammatory cytokines, an effect which is correlated to induction of HSF-1 and HSP70 and inhibition of NF-κB and STAT activation. Our findings demonstrate a significant therapeutic benefit of HT in controlling arthritis progression in a clinically relevant mouse model, with an efficacy similar to methotrexate. Mechanistically, HT targets highly relevant anti-inflammatory pathways which strongly support its increased study for use in clinical trials for RA.

## Introduction

Rheumatoid arthritis (RA) is an autoimmune disease of synovial joints. Excessive activation of innate immunity is an early event in RA and this appears to initiate synovial inflammation. Histological changes include pronounced angiogenesis and hyperplasia of the synovial membrane resulting from the infiltration of inflammatory cells. As the disease progresses, the formation of pannus, the locally invasive synovial tissue which grows to cover the articular cartilage, occurs with high expression of matrix metalloproteinases (MMPs) and is involved in the erosion of bone and cartilage.

Macrophages play a central role in the pathogenesis of RA. They accumulate in the synovial membrane and cartilage-pannus junction and secrete pro-inflammatory cytokines and chemokines which activate and recruit other inflammatory cells, driving a chronic cycle of damaging inflammatory responses [1, 2]. TNF-α is the principal pro-inflammatory cytokine produced by activated macrophages which functions synergistically with other cytokines, such as IL-1β, to stimulate synovial fibroblasts and chondrocytes to secrete enzymes that degrade proteoglycans and collagen, leading to tissue destruction. Although the underlying mechanisms of persistent macrophage activation are not entirely understood, pharmacological targeting of pro-inflammatory cytokine production by these cells has become an important strategy for preventing additional tissue damage and reducing painful symptoms of RA [3].

Conventional pharmacologic therapies for RA treatment include disease-modifying antirheumatic drugs (DMARDs), such as methotrexate (MTX) and biological agents that selectively block cytokines, such as anti-TNF-α antibodies. MTX, the most widely used DMARD, inhibits macrophage recruitment and proliferation, thereby reducing pro-inflammatory cytokine production [4]. Biological anti-TNF-α antibodies (i.e. Infliximab, Etanercept, Adalimumab) act to inhibit TNF-α function, reduce inflammatory cell influx into the joints and down-regulate synovial cytokine production. Furthermore, TNF-α blockade used in combination with MTX profoundly inhibits joint destruction even when clinical disease activity continues [5–7].

Although these drugs have been critical for understanding RA and controlling disease progression, their continuous use has been found to be highly immunosuppressive resulting in increased risk of bacterial and fungal infections [8, 9], limiting clinical use for many patients. Moreover, these treatments have been linked to increased risk of lymphoma and skin tumors in RA patients [10, 11]. Therefore, other approaches are necessary to reduce the necessary dose or toxicity of these treatments while maintaining or improving efficacy to control joint inflammation and damage.

Many arthritis patients turn to “traditional” therapies outside of their physician-prescribed pharmacological treatments, including heat treatment (HT), such as use of heat packs, mixtures of hot wax and mineral oil, balneotherapy and various bath therapies [12–14]. HT has long been recognized to reduce inflammation and ease some of the pain and stiffness of arthritis [15–17]. It is generally assumed that heat increases blood flow in the inflamed region, relaxing muscle activity and tension; however, whether this, or another immunological effect is the underlying basis for its effectiveness in reducing RA symptoms has never been demonstrated experimentally. In fact, there are remarkably few experimental explorations which may help define more precisely the efficacy of HT in animal models of arthritis. Without a more precise knowledge base of how HT affects RA pathology, there has been little impetus for clinically testing its potential benefits either alone or in combination with pharmacological therapies. In a single earlier study, Schmidt *et*. *al*. applied daily hyperthermia in different animal models of inflammation (including adjuvant arthritis, a RA model where rats were immunized with Complete Freund’s Adjuvant). They found that HT resulted in significant benefit and the authors hypothesized that HT may have anti-inflammatory and immunosuppressive effects [18], although mechanisms were not explored. Previous *in vitro* studies using macrophage cell lines or human monocyte-derived macrophages have shown that hyperthermia suppresses expression of pro-inflammatory cytokines including TNF-α, IL-6 and IL-1β [19, 20]. Studies published by our group also showed that systemic hyperthermia treatment not only affects tissue blood flow, but also modulates immune cell function and prevents another type of autoimmune disease in mouse models (type I diabetes) [21, 22].

Based on these studies, we tested here the hypothesis that mild heating reduces RA symptoms by reducing pro-inflammatory cytokine production in a clinically relevant murine model of collagen-induced arthritis (CIA). We also test effects of HT on molecular processes involving macrophage cytokine production and its efficacy in comparison to methotrexate, a well-studied drug used for the treatment of RA.

## Materials and Methods

### Ethics statement

BALB/c (NCI) and DBA/1J (The Jackson Laboratory) mice were maintained in specific pathogen-free facilities at Roswell Park Cancer Institute (RPCI, Buffalo, NY). All animal procedures were performed in strict accordance with the recommendations *in the Guide* for the Assessment and Accreditation of Laboratory Animal Care International. The protocol was approved by the Institutional Animal Care and Use Committee at Roswell Park Cancer Institute (Protocol number: 797M and 988M). For heat treatment, mice received saline to prevent dehydration. Mice body temperature was monitored every hour to prevent over-heating. Mice were euthanized by CO_2_ asphyxiation followed by cervical dislocation.

### Induction of collagen-induced arthritis (CIA)

Six-week-old DBA/1J female mice were immunized intradermally, at the base of the tail with 100 μg bovine collagen II (CII) emulsified in 50 μL complete Freund’s adjuvant (containing 1 mg/mL heat-killed *Mycobacterium tuberculosis* H37RA, Chondrex) on day 0 and with 50 μg bovine CII with 25 μL incomplete Freund’s adjuvant (Chondrex) on day 21. Animals were monitored regularly for swelling of paws, and a clinical score (0–3) was given for each paw. The clinical grade of the arthritis was determined using the following criteria: grade 0 (no swelling, no alteration in coloration of the paws), grade 1 (swelling or focal redness of finger joints), grade 2 (mild swelling of wrist or ankle joints) and grade 3 (severe swelling of the entire paw). The scores of all four paws were totaled and the incidence of CIA was calculated by dividing the number of mice showing disease symptoms of any paws by the total number of mice tested.

### Heat treatment (HT) and anti-rheumatic drug treatment protocol

For prophylactic studies, mice were randomized into treatment or control groups starting 22 days after immunization. Mice received HT for 6 hours, twice a week or HT for 30 minutes, 5 days a week for a total of 6–9 weeks. To reduce the risk of dehydration associated with heating, mice were injected intraperitoneally with 1 mL sterile saline prior to beginning treatment and immediately placed in microisolator cages preheated to 36.5°C in a gravity convection oven (Memmert model BE500, Wisconsin Oven). Mice core body temperatures were raised to 39.0°C (±0.2°C) within 20 min and then maintained for 30 minutes or 6 hours by adjusting the incubator temperature. Core body temperature in each cage was monitored with the Electronic Laboratory Animal Monitor System using mice that had microchip transponder (Bio Medic Data Systems) implanted. Non-HT control mice were kept at standard room temperature (approximately 22–23°C) and subjected to the same handling. For therapeutic studies, mice received HT (6 hours, twice a week) starting at day 35 when the mean disease severity score was about 2. For anti-rheumatic drug treatment, mice received intraperitoneal injection of MTX (0.1, 1 or 5 mg/kg, Sigma-Aldrich) 3 times a week for a total of 6–9 weeks.

### Histological analysis

Mice were euthanized and hind paws were removed, fixed in zinc (BD Biosciences) for 1 day and then transferred into decalcification buffer (containing Tris, KOH, EDTA, and polyvinylpyrolidone) for 2 weeks. After decalcification, the specimens were processed for paraffin embedding. Tissue sections were stained with hematoxylen and eosin and blinded assessed for histological features.

### Single cell suspensions from paws for cell infiltration

Paws were collected from naïve or arthritic mice. Single cell suspension was prepared by digestion with collagenase IV (10 mg/mL, Worthington Biochemical corp) in RPMI at 37°C for 3 hours. Cells were filtered and incubated with mAbs specific for CD4, CD8, CD11b (BD Biosciences) and CD11c (BioLegend) and analyzed by flow cytometry (BD Biosciences). Analysis of data was performed with FCS Express (De Novo Software).

### IFN-γ production by collagen II-specific T cells in lymph nodes (LN)

Inguinal, lumbar and mesenteric LNs and spleen were harvested and single cell suspensions were prepared. LN cells (6 × 10^5^) were co-cultured with total splenocytes (1.2 × 10^5^) as the APCs and 100 or 50 μg/mL of type II collagen for 72 hours. Supernatants were collected and analyzed for the presence of IFN-γ by ELISA (BD Biosciences).

### ELISA assay for serum TNF-α and anti-collagen II antibodies

Sera were collected from the blood of the retroorbital venous plexus sequentially at various times to measure TNF-α (BioLegend) and CII-specific antibodies by ELISA. For anti-CII antibodies, ELISA plates were coated with bovine CII (5 μg/mL) at 4°C overnight, and then blocked with PBS containing 10% FBS. Serum samples (1:25000 and 1:100000) were added and then followed by biotin-labeled anti-mouse IgG, IgG1, IgG2a and IgG2b antibodies (BioLegend). Color reaction was induced by TMB (BD Biosciences) and read the absorbance at 450 nm.

### Preparation of tissue homogenates from mouse paws

Protein extracts of mouse paws from naïve or CIA mice were prepared in CellLytic MT Mammalian Tissue Lysis buffer (Sigma-Aldrich) with Protease inhibitor Cocktail (Thermo Scientific).

### LPS administration, peritoneal macrophages isolation and in vitro stimulation

BALB/c mice were injected intraperitoneally with 10 μg LPS (Sigma-Aldrich) in 1 ml sterile saline. Peritoneal macrophages were harvested 3 days post LPS injection by peritoneal lavage with 6 mL cold PBS and collected by centrifugation. Macrophages were enriched by adherence to plastic, recovered overnight and re-stimulated *in vitro* with 100 ng/mL LPS and 25 U IFN-γ (R&D systems) at 37°C or 39.5°C for indicated time points. Supernatant was collected for the presence of TNF-, IL-1 (Biolegend), IL-6 (BD Biosciences) and HSP70 (R&D systems) by ELISA. In some experiments, macrophages were treated with HSP70 inhibitors (KNK437, EMD chemicals; Pifithrin-μ, Sigma-Aldrich) together with LPS/IFN-γ.

### Measurement of NF-κB nuclear translocation

Peritoneal macrophages collected from LPS-challenged mice were re-stimulated with LPS and IFN-γ at 37°C or 39.5°C for 30 minutes and then incubated with mAbs for mouse CD11b (BD Biosciences) and fixed with 4% formaldehyde (Polysciences, Inc). The cells were incubated with anti-NF-κB p65 Ab (Santa Cruz biotechnology) in permeabilization buffer and analyzed by ImageStream flow cytometry (Amnis). Anti-DRAQ5 DNA dye (Cell Signaling) was used for nuclear staining. NF-κB nuclear translocation was quantified by calculating the similarity score (SS) of the NF-κB and nuclear dye DRAQ5 images in CD11b^+^ macrophages. Low SS show no correlation (predominant cytoplasmic distribution of NF-κB) and high SS show a positive correlation (predominant nuclear distribution of NF-κB). The relative shift in the distribution between two populations was calculated using the Fisher's Discriminant ratio (*R*_d_ value) [23] where R_d_ = (SS of treated-untreated)/(standard deviation of the SS from treated+ untreated). Higher *R*_d_ value indicated more NF-κB was translocated into the nucleus.

### Chromatin immunoprecipitation (ChIP) assay

Peritoneal macrophages were re-stimulated with LPS/IFN-γ at 37°C or 39.5°C for 1 hour, cross-linked with 1% formaldehyde and collected by centrifugation. The cell pellets were resuspended in SDS lysis buffer with protease inhibitors (Thermo Scientific) and sonicated for five 10 seconds burst using Sonic Dismembrator model 300 (Fisher). Sonicated cell lysates were precipitated with 5 μg anti-NFκB p65 (Abcam) or species and isotype matched control antibody (normal rabbit IgG, Cell Signaling) at 4°C overnight. The immune complexes were collected with ChIP-grade protein G magnetic beads (Cell Signaling). Cross-linked protein-DNA was then eluted and reverted by incubating at 65°C overnight. DNA was extracted, used as template for quantitative real-time PCR and then analyzed using an Applied Biosystems real-time PCR system (ABI 7900 HT). Data were generated with the comparative threshold cycle (ΔΔCT) method [24] and normalized to the input and control IgG. The following primers were used for TNF promoter:

-912/-763: forward, gagaagtgactccactggagggt; reverse, actgcggtacatcaactcagacat;
-685/-543: forward, aaggcttgtgaggtccgtga; reverse, aagtggctgaaggcagagca;
-586/-468: forward, acttcccaactctcaagctgctct; reverse, gtgcttctgaaagctgggtgcata;
-364/-182: forward, tctggaggacagagaagaaatg; reverse, ggtttggaaagttggggacac.

### Western blotting analysis

Cell extracts were prepared in lysis buffer (containing 0.5 M Tris, 2.5 M NaCl, 500 mM NaF and 10% nonionic P_40_ with protease inhibitors: 200 mM Na_3_VO_4_, 0.5 M β-glycerophosphate, 0.25 M NaPPi, 0.1 M PMSF, 1 mg/mL leupeptine, 0.1 M benzamidine and 1 mg/mL aprotinin). Protein concentration was measured using protein assay dye reagent (Bio-Rad) according to the manufacturer’s instructions. For Western blotting analysis, 40 μg total protein lysates were resolved by SDS-PAGE, transferred to a polyvinylidene difluoride membrane (Millipore) and blocked with either 5% nonfat milk in PBS-T or 3% BSA in TBS-T. The membrane was then probed with primary antibodies followed by horseradish peroxidase-conjugated secondary antibody and developed with ECL-detecting reagents (Thermo Scientific) and autoradiography film. The band intensity was quantified using Scion Image (Scion Corporation). The following primary antibodies were used: phospho-IKK, phospho-IκB, phosphor-STAT1, STAT1, Mcl-1, Bcl-2, Bcl-xL, caspase-3 (Cell Signaling), HSP70 (Thermo Scientific), HSF-1 (Stressgen), NFκB p65, p50 (Santa Cruz Biotechnology) and HIF-1α (Novus Biologicals).

### RNA extraction and quantitative real-time PCR

Peritoneal macrophages or mouse paw tissues were lysed and total RNA was extracted using the RNeasy kit (QIAGEN). One microgram of RNA was reverse transcribed using oligo (dT) and Superscript III first strand synthesis system (Invitrogen). Resulting cDNA was used as template to analyze the expression of target genes by quantitative real-time PCR using specific primers and SYBR green master mix (Roche). The results of real time-PCR were then analyzed using an Applied Biosystems real-time PCR system (ABI 7900 HT). Data were generated with the comparative threshold cycle (ΔΔCT) method and normalized to the housekeeping gene GAPDH. The following primers were used: TNF-α forward, caccacgctcttctgtctactgaact; reverse, IL-6 forward, gccagagtccttcagagagatacag; reverse, cccaacattcatattgtcag; IL-1β forward, agctctccacctcaatggacagaa; reverse, attgcttgggatccacactctcca; IL-10 forward, ctgaggcgctgtcatcgatttctc; reverse, tggccttgtagacaccttggtctt; gggctacaggcttgtcactcgaattt; HSP70 forward, agcgaggctgacaagaagaaggt; reverse, accctggtacagcccactgatgat; MCP-1 forward, tggctcagccagatgcagt; reverse, ttgggatcatcttgctggtg; MIP-1α forward, caaccaagtcttctcagcgccata; reverse, ggagcaaaggctgctggtttcaa; RANTES forward, tgcagtcgtgtttgtcactcgaag; reverse, tagagcaagcaatgacagggaagc; MIP-2 forward, acaaaggcaaggctaactgacctg; reverse, ataagtgaactctcagacagcgaggc; CCR1 forward, gcccagaaacaaagtctgtgtgga, reverse, tccatcctttgctgaggaactggt; CCR2 forward, agaagaggcattggattcaccaca; reverse, tgccgtggatgaactgaggtaaca; CCR3 forward, acacctatgaatatgagtgggcacc, reverse, atgaacaccagggagtacagtgga; CCR4 forward, tcagaagagcaaggcagctcaact; reverse, tgtctgtgacctctgtggcattca; CCR5 forward, actctggctcttgcaggatggatt; reverse, tggcagggtgctgacataccataa.

### Statistical analysis

Arthritis disease severity between the treatment groups were analyzed with the non-parametric Mann-Whitney U-test and other data were analyzed with Student’s *t* tests and repeated-measures two-way ANOVA to determine statistical significance. *P* < 0.05 represents statistically significant differences.

## Results

### Significant reduction in arthritis disease severity and joint damage by heat treatment in a collagen-induced arthritis mouse model

**Fig 1:**
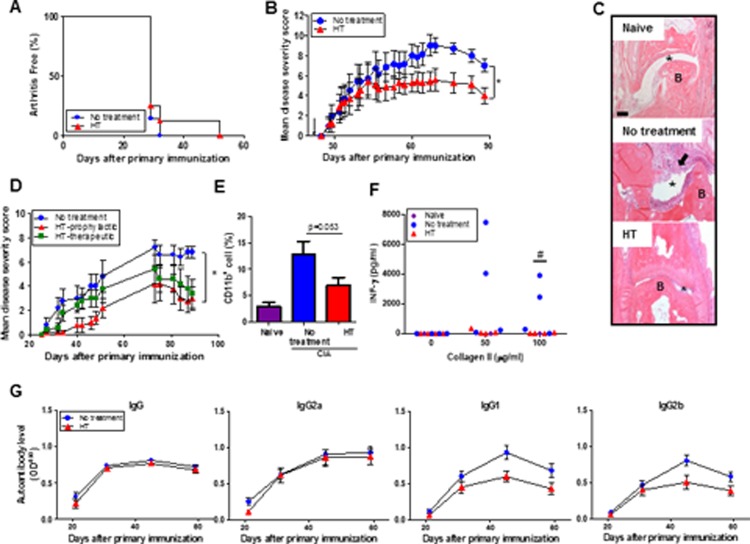
Heat treatment reduces arthritis disease severity and joint damage in a collagen-induced arthritis mouse model. (A-C), DBA1 mice were immunized with bovine CII and either received HT (6 hrs, 2x/week) or left untreated for total 9 weeks, beginning on day 22 (arrow). Data are presented as (A) incidence of arthritis in the indicated groups (n = 8) and (B) mean disease severity scores ± SEM (n = 7). (C), H&E-stained section of synovial toe joints from representative naïve and arthritic mice (day 88). Synovial hyperplasia and immune cell infiltration was shown by the arrow. B: bone; *: joint space. Scale bar, 100 μm. (D), For the prophylactic study, mice received HT from day 22, whereas in the therapeutic study, mice received HT starting at day 35 when the mean disease severity score reached 2. * p < 0.05, non-parametric Mann-Whitney U-test to compare treated to untreated mice. (E), Flow cytometric analysis of CD11b^+^ macrophage infiltration in the joints of naïve and CIA mice (day 88) (n = 4 for naïve and n = 9 for CIA mice). (F), LN cells were restimulated with bovine CII (0, 50, 100 μg/ml) for 72 hr. Supernatants were collected to determine the levels of IFN-γ by ELISA. Data shows individual mice. * p < 0.05, paired Student *t* test (G), Serum levels of anti-CII IgG, IgG1, IgG2a and IgG2b were detected by ELISA (n = 8). Error bars show SEM. Data are representative of three experiments. * p < 0.05, paired Student *t* test or repeated-measures two-way ANOVA to compare treated to untreated group.

In this study, we used the CIA model to evaluate the therapeutic potential of HT in controlling arthritis development. DBA/1J mice were immunized with bovine CII to develop CIA. Mice received 6 hr HT (6 hour twice a week) before visible symptoms occurred in a prophylactic protocol. CIA is a self-limiting disease, consisting of a joint damaging inflammatory phase followed by a remission phase in which there is a progressive decrease in disease activity. While HT had no effect on the onset of disease or on overall incidence (Fig. 1A), it caused a remarkable reduction in disease severity in the remission phase as compared to mice without HT (Fig. 1B). Histology confirmed the reduced tissue damage and immune cell infiltration into the joints of heat-treated mice. These mice had a similar articulation to that of naive mice. Conversely, joints of the non-treated arthritic mice revealed synovial hyperplasia and increased leukocyte infiltration at bone and cartilage interfaces as well as tissue destruction (Fig. 1C, black arrow). To test whether HT was effective in a therapeutic setting, mice received 6 hr HT when the mean disease severity score reached ~2. We observed HT significantly reduced disease severity in the prophylactic setting. In the therapeutic setting, 6 hr HT exerted no effect in the initial inflammatory phase (day 22–51) however, reduced disease severity was evident in the later remission stage of arthritis (day 73–89; Fig. 1D), in heated mice, although this did not quite reach statistical significance.

As macrophages are prominent in the inflamed joints of RA patients, we evaluated the impact of 6 hr HT on their presence within inflamed joints of CIA mice. We found CD11b^+^ cells (i.e., macrophages) but little or no T cells and dendritic cells in the inflamed joints of arthritic mice (S1A Fig.). There was an obvious trend for decreased numbers of CD11b^+^ macrophages within the paws of heated mice with CIA as compared to non-HT CIA mice (Fig. 1E). Furthermore, consistent with previous studies, there was a positive correlation between macrophage infiltration and disease severity (S1B Fig.).

To study the mechanisms contributing to the reduction of macrophages in inflamed joints of HT mice, we investigated the effect of 6 hr HT on expression of chemokines and chemokine receptors required for macrophage recruitment. We found up-regulation of MCP-1, RANTES, CCR1 and CCR2 in arthritic mice compared to naïve mice, while expression of MIP-1α, MIP-2, CCR3, CCR4 and CCR5 was similar. Moreover, HT had no effect on the expression of chemokine or chemokine receptors (S2A and S2B Figs.).

To investigate whether apoptosis in HT mice could account for decreased macrophages, we analyzed expression of Bcl-2 family proteins as well as activated caspase-3. Up-regulation of Bcl-xL, but not Mcl-1, Bcl-2 or caspase-3 was observed in the paws of arthritic mice compared to naïve mice (S2C and S2D Figs.), suggesting activation of pro-survival signals in the inflamed joints. However, there was no difference in Bcl-xL expression between HT and untreated arthritic mice.

### Heat treatment inhibits the production of IFN-γ by CII-specific T cells but has little effect on CII-specific antibody production

Collagen-specific T and B cells play an important role in arthritis pathogenesis by secreting cytokines including IFN-γ [25] and autoantibodies [26]. To assess the effects of 6 hr HT on these cellular responses, lymph node (LN) cells were isolated from arthritic mice and stimulated *in vitro* with bovine CII for IFN-γ production. We found that LN cells from untreated mice produced high levels of IFN-γ after stimulation, compared to HT and naïve mice (Fig. 1F). We next measured serum CII-specific antibodies, finding that levels of CII-specific IgG and IgG2a increased with disease progression; however, there was no difference between HT and untreated mice. CII-IgG1 and IgG2b peaked at day 45 and declined as the disease progressed. While there was a reduction in both CII-IgG1 and IgG2b at the peak time in HT mice, there was no difference in these Igs at later points in disease progression (Fig. 1G).

### Long duration and short daily heat treatments are as effective as methotrexate in reducing arthritis disease severity

**Fig 2:**
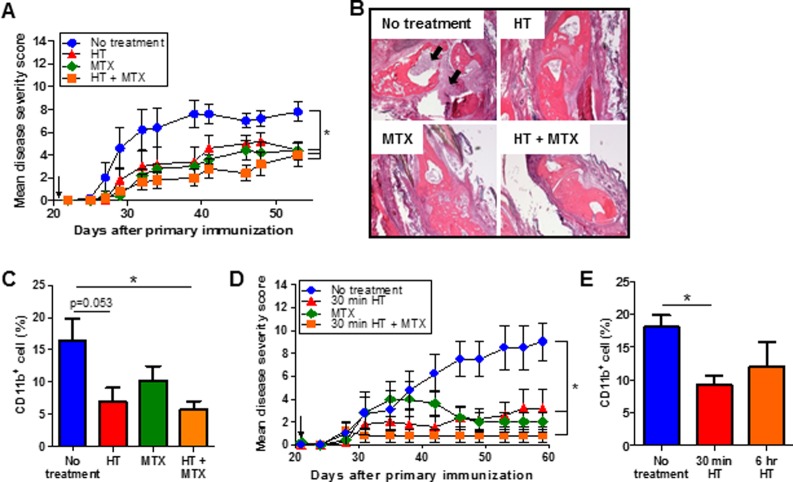
Effects of heat treatment and combination therapy with methotrexate in controlling the development of arthritis. (A-C), DBA1 mice were immunized with bovine CII and received HT (6 hrs, 2x/week), MTX (5 mg/kg, 3x/week), HT in combination with MTX or left untreated for total 5 weeks, beginning on day 22 (arrow). (A), Data are presented as mean disease severity scores ± SEM (n = 5). (B), H&E-stained section of synovial joints from arthritic mice with different treatments (day 53). Synovial hyperplasia and immune cell infiltration was shown by the arrows. (C), Flow cytometric analysis of CD11b^+^ macrophage infiltration in the joints of CIA mice with different treatments (day 53). (D), DBA1 mice were immunized with bovine CII and received short daily 30-minute HT, MTX (5 mg/kg, 3x/week) or HT in combination with MTX from day 22. Data are presented as mean disease severity scores ± SEM (n = 5). (E), Flow cytometric analysis of CD11b^+^ macrophage infiltration in the joints. * p < 0.05, non-parametric Mann-Whitney U-test (A and D) and paired Student *t* test (C and E) to compare treated to untreated group.

Observing that 6 hr HT decreased disease severity led us to compare its effectiveness with MTX, a pharmacological anti-rheumatic drug. Different doses of MTX (0.1, 1 and 5 mg/kg) were used alone or in combination with 6 hr HT. Low doses of MTX (0.1 and 1 mg/kg) had little effect in controlling disease progression. However, HT alone or in combination with MTX (1 mg/kg) reduced arthritis progression (S3 Fig.). High dose MTX (5 mg/kg) effectively reduced disease score and there was no additional benefit by combining with HT (Fig. 2A). While significant histological abnormalities were seen in untreated mice (Fig. 2B, black arrows), only minor tissue damage and synovial hyperplasia was seen in the finger joints with HT and MTX treatment. We also observed less CD11b^+^ macrophage accumulation in inflamed joints of CIA mice treated with both HT and MTX as compared to the untreated mice (Fig. 2C).

To determine whether a different HT protocol could be effective in reducing RA symptoms, we tested the therapeutic efficacy of short daily HT (30 minutes, 5 times a week) with MTX in controlling arthritis disease severity. We demonstrated that both short daily HT and MTX significantly reduced arthritis disease scores as compared to untreated mice (Fig. 2D). Again combination of short daily HT and MTX resulted in a more potent benefit than treatment with MTX alone. Short daily HT significantly reduced CD11b^+^ macrophage accumulation in inflamed joints (Fig. 2E).

### Heat treatment reduces inflammation and promotes anti-inflammatory mediators

**Fig 3:**
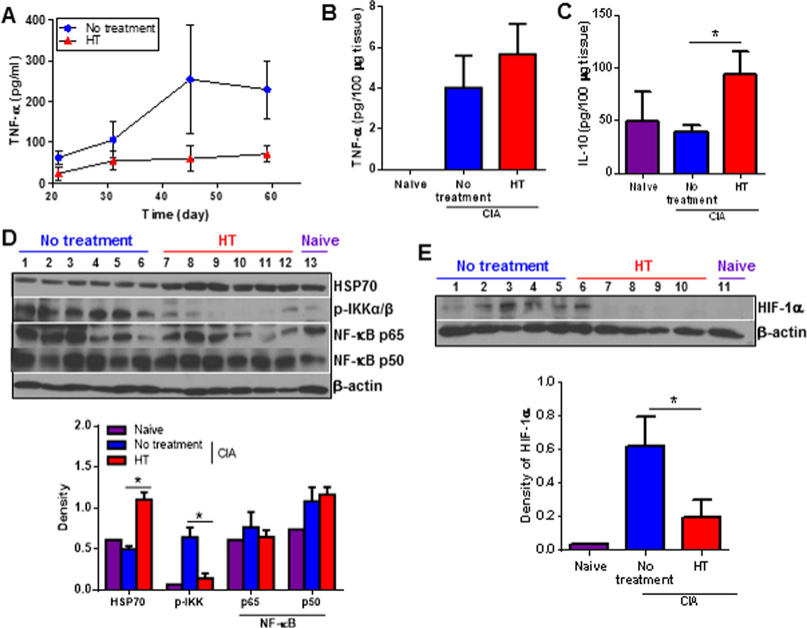
Multiple targets are affected by heat treatment. (A), Serum TNF-α concentration from CIA mice was determined by ELISA. Error bars show SEM (n = 7). (B-C), TNF-α and IL-10 concentrations were detected in the tissue homogenates from naïve and CIA mice paws by ELISA (day 53). Error bars show SEM. Data are representative of two experiments. (D-E), tissue homogenates were prepared from naïve and CIA mice paws and expression of phosphorylated IKKα /β, NF-κB p65, p50, HSP70 and HIF-1α were detected by Western blotting. Each lane represents different mice. The graph shows the ratio of the band intensity of proteins normalized to β-actin. * p < 0.05, paired Student *t* test to compare treated to untreated group.

TNF-α is directly involved in the pathogenesis of arthritis sustaining joint inflammation and cartilage destruction [27]. To determine whether 6 hr HT modulated TNF-α production in the prophylactic model, we collected serum from HT and untreated mice throughout disease progression. We observed less TNF-α levels in HT versus untreated mice (Fig. 3A). Tissue TNF- α levels in inflamed joints were very low at the end of the study (day 53) in all groups (Fig. 3B). However, levels of anti-inflammatory IL-10 were increased in HT mice (Fig. 3C).

Heat stress is an important stimulus for the induction of heat shock proteins (HSPs) [28], particularly HSP70 which plays an anti-inflammatory role by preventing NF-κB activation and down-regulating inflammatory gene expression [29]. We wondered whether 6 hr HT induced HSP70 expression which could be involved in modulation of cytokine production by macrophages in CIA. To test this, paw homogenates from 6 hr HT, untreated arthritic mice as well as naïve controls were analyzed for HSP70 expression. We identified an up-regulation of HSP70 in inflamed joints from HT arthritic mice compared to untreated arthritic and naïve mice (Fig. 3D). We next asked if 6 hr HT affected NF-κB activation by determining IKK phosphorylation and NF-κB protein expression. We found increased IKK phosphorylation in untreated arthritic mice compared to naïve mice (Fig. 3D), indicating enhanced NF-κB activation with CIA. HT inhibited IKK phosphorylation. There was no change in total NF-κB p65 and p50 proteins in naïve or arthritic mice (Fig. 3D), demonstrating that 6 hr HT reduced NF-κB activation without affecting their expression levels.

The inflamed RA microenvironment has a marked increase in hypoxia [30] and consistent with this observation, abundant HIF-1α expression in synovial macrophages has been demonstrated [31]. We wondered whether 6 hr HT could modulate the hypoxic environment of arthritic joints and affect macrophage function. While the expected increase in HIF-1α occurred during arthritis development, HT significantly reduced the HIF-1α up-regulation (Fig. 3E).

### *In vitro* heating targets cytokine production and heat shock protein expression in activated macrophages

**Fig 4:**
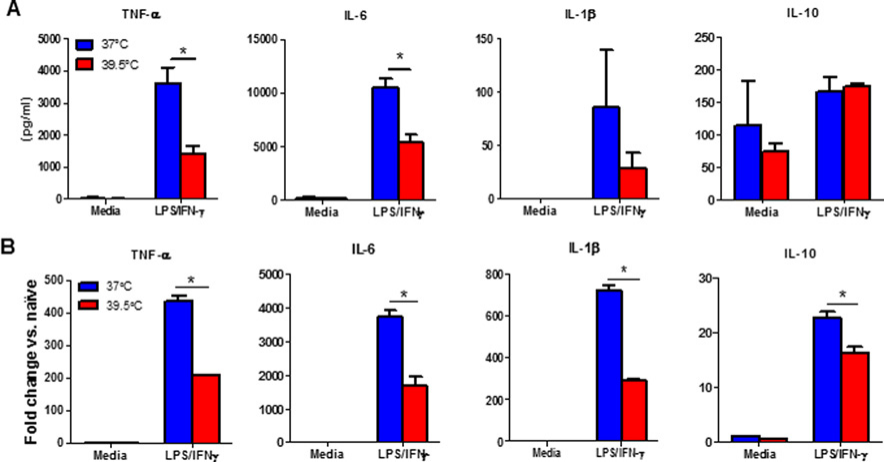
In vitro heat treatment inhibits LPS-induced cytokine production by activated macrophages. (A-B), BALB/c mice were injected intraperitoneally with 10 μg of LPS. Peritoneal macrophages were harvested 3 days post LPS injection, recovered overnight and re-stimulated (2x10^5^/well) with LPS (100 ng/mL) and IFN-γ (25 U) at 37°C or 39.5°C for 6 hours to determine TNF-α, IL-6, IL-1β and IL-10 production by ELISA (A) or re-stimulated (1x10^6^/well) for 4 hours to measure TNF-α, IL-6, IL-1β and IL-10 mRNA expression by quantitative real-time PCR (B). The results are presented relative to GAPDH and baseline expression in unstimulated cells at 37°C. Cells from each treatment condition were pooled from 2–4 mice and measured in triplicate. Data are mean ± SD. Data are representative of three independent experiments. * p < 0.05, paired Student *t* test and repeated-measures two-way ANOVA.

Because the number of macrophages which can be isolated from the inflamed, damaged tissues of arthritic mice is quite limited, we used LPS-activated macrophages to study in greater detail the mechanisms by which HT impacts macrophage function. We isolated activated macrophages from BALB/c mice 3 days post LPS challenge. Cells were re-stimulated *in vitro* with LPS and IFN-γ at either 37°C or 39.5°C and pro- and anti-inflammatory cytokine production was measured using a commercial ELISA kit. Heating macrophages resulted in reduced TNF-α and IL-6 following LPS/IFN-γ re-stimulation compared to the cells maintained at 37ºC (Fig. 4A), which was consistent with previous studies that hyperthermia had anti-inflammatory effects by suppressing activated macrophage pro-inflammatory cytokine expression [19, 20, 32]. IL-1β production was decreased in heated cells although it did not reach a statistical significance. LPS/IFN-γ induced low IL-10 production by macrophages in heat-treated and control groups (Fig. 4A). We confirmed that the thermally-mediated-inhibitory effect on macrophage pro-inflammatory cytokine production occurred at the transcriptional level (Fig. 4B), which has been shown by Ensor et al. that thermally-inhibition of pro-inflammatory cytokine expression may be linked to a marked reduction in cytokine gene transcription and mRNA stability [33]. These data suggested that *in vitro* heating inhibited activated macrophage production of pro-inflammatory cytokine, which was consistent with previous in vitro studies and our *in vivo* findings in the arthritic mice.

**Fig 5:**
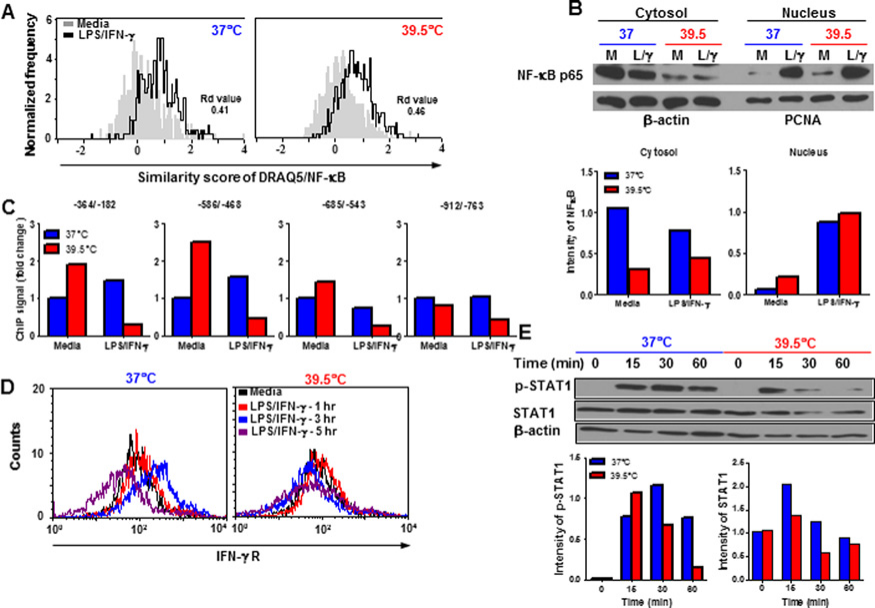
Effects of *In vitro* heat treatment on macrophage NF-κB and STAT1 activation. (A-B), Peritoneal macrophages were isolated from LPS-challenged mice, recovered overnight and re-stimulated (1x10^6^/well) with LPS/IFN-γ at 37°C or 39.5°C for 30 min. (A) These cells were then stained with antibodies against CD11b, NF-κB p65 and DRAQ5 DNA dye and then analyzed by ImageStream flow cytometry. CD11b^+^ cells were gated to show the SS between DRAQ5 nuclear staining and NF-κB staining or (B) nuclear and cytosol proteins were isolated to detect NF-κB p65 expression by Western blotting. The graph shows the ratio of the band intensity of NF-κB p65 normalized to β-actin or PCNA. Data are representative of two independent experiments. (C) Macrophages were stimulated at 37°C or 39.5°C for 1 hour. Cross-linked chromatin was immunoprecipitated with anti-NF-κB p65 antibody and analyzed for NF-κB p65 binding to the TNF-α promoter region by quantitative real-time PCR with primers spanning the regions-364/-182, -586/-468, -685/-543 and-912/-763. Fold change is normalized to the input and control IgG and then compared with unstimulated cells at 37°C. Cells from each treatment condition were pooled from 4 mice. Data are representative of two independent experiments. (D-E), Macrophages were re-stimulated with LPS/IFN-γ at 37°C or 39.5°C for indicated times. (D) Cells were stained with CD11b and IFN-γ receptor antibodies. CD11b^+^ cells were analyzed for expression of IFN-γ receptor by flow cytometry. (E) Cell lysates were prepared to detect phosphorylated STAT1 and total STAT1 by Western blotting. Quantification of the band intensity of pSTAT1 and STAT1 is normalized to β-actin. Cells from each treatment condition were pooled from 4 mice. Data are representative of two independent experiments.

NF-κB plays an important role in inflammatory responses by regulating pro-inflammatory cytokine production. To assess whether heating modulated NF-κB activation in macrophages, we used ImageStream flow cytometry and Western blotting to measure NF-κB nuclear translocation. We found that *in vitro* heating did not affect LPS-induced NF-κB nuclear translocation (Fig. 5A-B). Next, we asked whether the binding of NF-κB to the TNF-α promoter was affected by heating. The murine TNF-α promoter contains 4 κB binding sites located at 210, 510, 655 and 850 nucleotide upstream of the transcription start site [34]. Previous studies by Cooper et al. showed that fever-range temperatures selectively reduced LPS-induced recruitment of NF-κB transcription factor to the TNF-α promoter regions [35, 36]. To test this, we found that *in vitro* heating resulted in a less NF-κB binding to the four κB sites in the TNF-α promoter region following LPS/IFN-γ re-stimulation (Fig. 5C) suggesting that *in vitro* heating influenced NF-κB signaling by inhibiting binding to the TNF-α promoter. These results correlated well with our findings in the CIA model that HT inhibits NF-κB activation.

IFN-γ plays a key role in macrophage activation by binding to cell surface receptors and signaling through the Janus kinase (JAK)-signal transducer and activator of transcription (STAT) pathway [37]. To determine whether HT modulated IFN-γ /STAT signaling, we analyzed IFN-γ receptor expression on the macrophage surface. At 37°C, IFN-γ receptor was up-regulated after 1 and 3 hours of stimulation with LPS/IFN-γ and then down-regulated after 5 hours of stimulation. When heated to 39.5°, LPS/IFN-γ-induced up-regulation of IFN-γ receptor was blocked (Fig. 5D). Furthermore, heating reduced STAT1 phosphorylation and also inhibited total STAT1 expression (Fig. 5E) demonstrating that heating macrophages modulated LPS/IFN-γ-mediated STAT signaling as another mechanism regulating macrophage activation and cytokine production.

**Fig 6:**
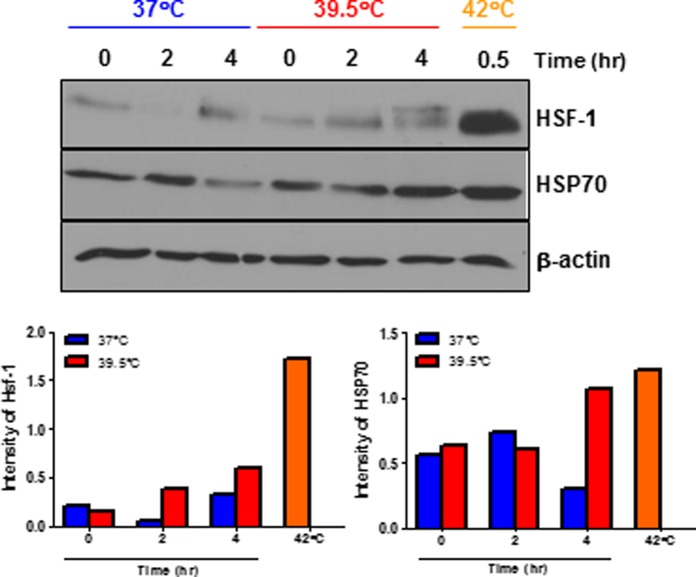
*In vitro* heat treatment increases HSF-1 and HSP 70 expression which may regulate TNF-α production in activated macrophages. Peritoneal macrophages were harvested from LPS-challenged mice, recovered overnight and re-stimulated with LPS (100 ng/mL) and IFN-γ (25 U) at 37°C or 39.5°C for indicated times. Cell lysates were prepared from these cells to detect HSF-1 and HSP70 by Western blotting. Cells stimulated at 42°C for 30 min were used as positive controls. The graph shows the ratio of the band intensity normalized to β-actin. Data are mean ± SEM. Data are representative of two independent experiments. * *p* < 0.05, paired Student *t* test.

Last, we observed an induction of heat shock factor 1 (HSF-1, a transcription factor for HSPs) in a time-dependent manner and there was also a small increase in HSP70 at 4 hour time point in heated macrophages (Fig. 6), suggesting HT-induced HSF-1 and HSP70 may exert anti-inflammatory effects and contribute to the thermally-suppressed TNF-α production in macrophages.

## Discussion

Various pro-inflammatory cytokines are functionally active in synovial tissues and are associated with the pathogenesis of RA. Understanding the damaging role of these cytokines in RA has resulted in targeted therapeutic interventions, including anti-TNF-α, IL-1 and IL-6 antibodies. Other pharmacological approaches include methotrexate (MTX), which inhibits macrophage recruitment, proliferation and pro-inflammatory cytokine production. Although these therapies achieve very successful clinical improvement, toxic side effects, including impaired host defense, are serious, dose limiting problems for many patients. Moreover, strong resistance to drugs occurs in a high proportion of RA patients. Therefore, continuous development of new or improved therapies that can be used to either delay the administration of pharmacological drugs, to use in combination to reduce toxic side effects are important for further improvement of RA therapy. Preclinical models are critical to achieving this goal. Here, we assessed a very old therapy using modern experimental techniques to assess mechanism as well as efficacy using a CIA mouse model, which has often been considered a “gold standard” *in vivo* mouse model for RA studies since it shares many pathological similarities with human RA and is similarly strongly correlated with MHC class II gene expression [38]. However, while this is a valuable model, it does exhibit an important difference from human RA related to the course of disease and this must be taken into consideration as these data are extrapolated to potential human studies. CIA is a more self-limiting disease, consisting of a joint damaging inflammatory phase followed by a remission phase in which there is a progressive decrease in disease activity. In contrast, human RA is a chronic disease with a sustained inflammation for many years [39–41]. Nevertheless, although CIA lacks of chronic disease stage, it is still suitable for the studies of anti-inflammatory therapeutic strategies and has been used extensively for preclinical testing of therapies which were eventually tested in humans.

In our studies, a mild elevation of body temperature of mice with CIA was observed to result in significantly reduced arthritis disease progression in the prophylactic settings, but was less effective in the therapeutic settings, which is consistent with previous findings. Importantly however, the anti-inflammatory effects of HT (both a longer biweekly and short daily HT) were remarkably comparable to those of MTX, indicating that HT may work in a similar manner to pharmacological drugs used to treat arthritis. Thus, with additional clinical testing, a prescribed heating protocol for newly diagnosed RA patients could be highly feasible both at home and in the clinic, and could be accomplished by existing thermal therapy methods. With more detailed studies to determine optimal scheduling and duration of heat treatments, we may be able to delay and/or reduce the use of drugs such as MTX or other pharmacological arthritis treatments, which could delay development of drug resistance while reducing toxic side effects. These additional studies would certainly need to address whether local heating or regional/systemic heating protocols are the most effective. While patients are using both local (i.e., hot wax) as well as systemic (hot baths) approaches, the murine studies presented here have been exclusively systemic, due in large part to the difficulty of performing local heating of the joints of mice without use of restraints combined with full anesthesia, a condition which substantially interferes with more natural thermoregulatory neuro-vascular responses seen in non-anesthetized patients or mice.

Macrophage numbers in inflamed joints is useful as a biomarker for therapeutic efficacy [42] and our studies showed that macrophages may be a common target of both heat and drugs such as MTX. We observed decreased CD11b^+^ macrophage infiltration after HT which might be due to reduced macrophage recruitment or increased apoptosis. However, in later remission stages of disease, HT did not affect the expression of chemokines and chemokine receptors required for macrophage recruitment or the levels of apoptosis-related proteins. Therefore, HT might affect macrophage recruitment or cell survival in the earlier inflammatory phase of arthritis or HT might exert effects on macrophage migration. Thermally-reduced arthritis progression was also correlated with reduced CII-specific T cell activation measured by IFN-γ production but had no effect on CII-specific antibody production suggesting that HT may act differently in T and B cell function. These data indicated that changes in synovial macrophage infiltration can be used as a marker for therapeutic efficacy of heat treatment.

Previous studies have clearly demonstrated that inflammatory cytokines are essential for the development of RA and one important feature of RA is the imbalance between pro-inflammatory and anti-inflammatory cytokines in the joints [43]. Although the anti-inflammatory cytokines IL-10, IL-11 and IL-1RA are expressed by synovial mononuclear cells, they are not present in sufficient local concentrations to mediate their regulatory activity against the dominant pro-inflammatory cytokines [44]. In this study, we observed that HT altered the balance between pro and anti-inflammatory cytokines, as particularly seen by reduced serum TNF-α levels, which was similar to previous studies on MTX [45–48]. In addition, HT increased IL-10 production in inflamed joints, whereas TNF-α expression was unaffected demonstrating that TNF-α may be crucial at the onset of the disease but appeared less dominant at later remission stages. The regulatory role of IL-10 in RA has been confirmed in several CIA studies and is responsible for disease remission. Treatment of arthritic mice with recombinant IL-10 or adenoviral vectors expressing IL-10 results in suppression of CIA [49, 50]. Recent studies have also shown that the involvement of IL-10 in ameliorating the pathogenesis of CIA by inhibiting IL-17 production in macrophages and repressing classically activated M1 macrophages polarization [44]. Therefore, HT-increased IL-10 production in the joints may exert more potent regulatory effects in dampening CIA disease progression, which is more clinically relevant. In exploring further molecular mechanisms by which HT reduced disease progression, we observed decreased IKK phosphorylation in the joints of HT mice. Previous murine studies have shown that administration of dominant negative IKKβ or IKKβ inhibitor reduces joint inflammation and bone destruction [51, 52]. Therefore, HT might reduce joint inflammation by reducing IKK function and subsequent NF-κB activation. HSP70 has been shown to interact with IKK and prevent IκB phosphorylation and NF-κB activation [29]. Thus, our finding of HSP70 up-regulation in inflamed joints after HT suggested that HT may influence arthritis progression through HSP70. However, although total expression level of NF-κB p65 and p50 was not changed, we cannot rule out the possibility of HT promoting NF-κB p50/p50 homodimer formation which would repress NF-κB p65/p50 heterodimer DNA binding. Previous studies using macrophage cell lines [19, 20] and a moderate hyperthermia temperature (40°C) have provided strong evidence that activated HSF1 primarily represses TNF-α transcription [53, 54] while stimulating IL-10 activation [55]. If this occurs in the heat exposed mice tested here it could also explain, as a secondary set of events, reduced HIF-1α levels and reduced NF-κB activation, both of which can profoundly influence immune cells activity. In this study, we isolated activated primary macrophages from mice with LPS-induced inflammation and found that heating reduced pro-inflammatory cytokine production but did not affect NF-κB nuclear translocation. These results differed from data previously reported using macrophage cell lines [33, 35]. Cooper et al. showed that febrile-range temperature repressed TNF-α production while increased IL-1β expression by differentially modifying NF-κB and HSF-1 recruitment to cytokine gene promoters. Mouse IL-1β promoter lacked an intact HSF-1 binding site that is present in the human promoter. However, we observed reduced IL-1β RNA expression in heated macrophages. Evidence have shown that primary macrophages and cell lines exhibit different cytokine expression profiles upon LPS stimulation [56]. Therefore, whether primary macrophages and cell lines respond differently to HT and have different gene expression profiles need to be further confirmed. We also observed that heating reduced NF-κB binding to the TNF- α promoter region as well as IFN-γ/STAT signaling. Last, we observed that HT induced HSF-1 and HSP70 expression in macrophages. HSF-1 has been shown as the primary transcriptional modifier (as developed in earlier studies using cell lines [53–55]) resulting in reduced TNF-α, rather than HSP70 as the transcriptional regulator. Clearly, while much more work is needed to determine how these data compare to thermally induced changes in the macrophages found in RA affected tissues, it is possible that heating may target some of the same molecular pathways as novel pharmacological interventions.

Hypoxia is another hallmark of RA affected joints and must be considered in any potential model for how hyperthermia influences tissues. Macrophages accumulate in the hypoxic environment and respond rapidly to altered gene expression via up-regulation of HIF-1 and 2 [57]. Our results show that HT reduced HIF-1α expression in inflamed joints. HIF-1α can be activated in response to hypoxia and a number of non-hypoxic stimuli. NF-κB has been reported to play a role in hypoxia-induced HIF-1α mRNA expression. Since there is a significant level of cross talk between HIF-1α and NF-κB [58], it is possible that in the joints of HT mice, up-regulation of HSP70 could inhibit NF-κB activation in turn inhibiting HIF-1α expression. However, more research is needed to identify the link among HSP70, NF-κB and HIF-1α expression.

In summary, it is clear that temperature shifts in arthritic mice is associated with highly specific effects which can reduce the symptoms of arthritis. With increased study of thermally sensitive targets in inflamed joints, we could propose rationale therapeutic approaches that utilize heat alone, and/or in combination with other medications. With the benefits of low cost, safety and feasibility, a combination approach can possibly lesson the exposure of patients to the immune suppressive side effects of anti-TNF or other drugs currently prescribed for RA patients.

## Supporting Information

S1 Fig Effect of heat treatment on immune cell infiltration in the inflamed joints. (A), Flow cytometric analysis of CD11b, CD11c, CD4 and CD8 cell infiltration in the joints of CIA mice (day 88). Data are presented from a representative mouse. (B), Correlation between the percentage of CD11b^+^ macrophage infiltration in the joints and arthritis disease score. Each symbol represents an individual mouse.
(TIF)

S2 Fig Effect of heat treatment on the expression of chemokine/chemokine receptors and apoptosis-related proteins in the inflamed tissues. (A-B), RNA was isolated from the joints and mRNA level of chemokines MCP-1, MIP-1α, RANTES, MIP-2 (A) and chemokine receptors CCR1, CCR2, CCR3, CCR4, CCR5 (B) were analyzed by quantitative real-time PCR. The results are presented relative to GAPDH and naïve mice. Baseline expression in naïve mice was shown by the dash line. (C-D), Tissue homogenates were prepared from naïve and CIA mice paws and expression of Mcl-1, Bcl-2, Bcl-xL, caspase-3 and cleaved caspase-3 were detected by Western blotting. Each lane represents different mice. The graph shows the ratio of the band intensity of proteins normalized to β-actin.
(TIF)

S3 Fig Effect of heat treatment alone or combination with different doses of methotrexate in controlling the development of arthritis. (A to B), DBA1 mice were immunized with bovine CII and received heat treatment (6 hour, 2x/week), MTX (0.1 or 1 mg/kg, 3x/week), heat treatment in combination with MTX or left untreated from day 22. Data are presented as mean disease severity scores ± SEM from 5 individual mice. * p < 0.05, non-parametric Mann-Whitney U-test to compare treated to untreated mice.
(TIF)
