# Supplementary figures and images for: Defining Immunological Impact and Therapeutic Benefit of Mild Heating in a Murine Model of Arthritis
Source: PLoS One. 2015 Mar 20;10(3):e0120327. doi: 10.1371/journal.pone.0120327 (PMC4368208; doi:10.1371/journal.pone.0120327)

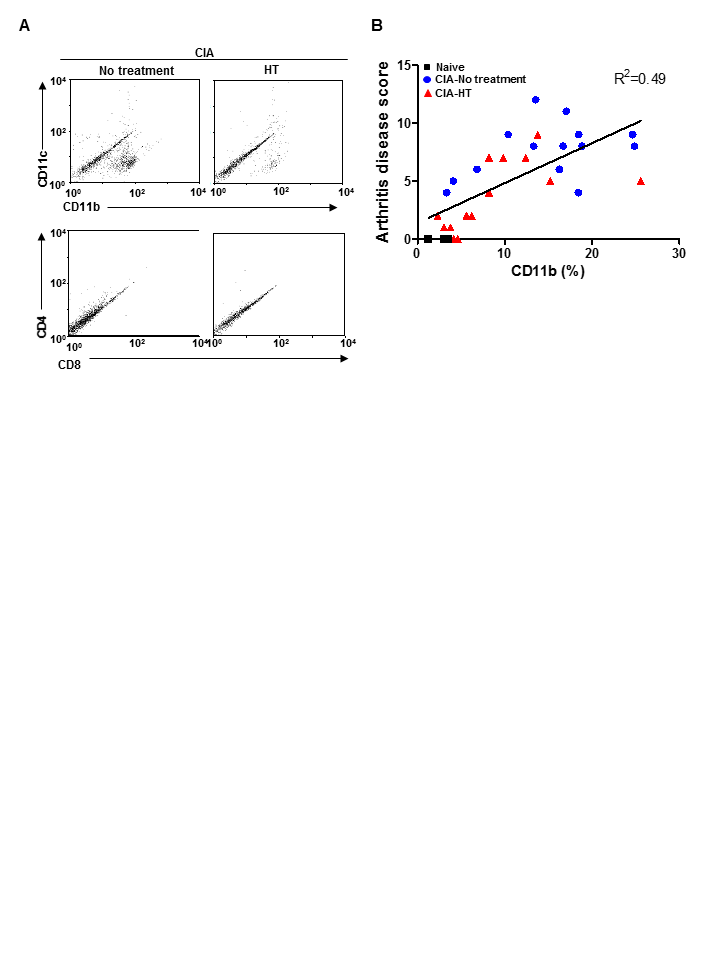

Supplement: S1 Fig — (A), Flow cytometric analysis of CD11b, CD11c, CD4 and CD8 cell infiltration in the joints of CIA mice (day 88). Data are presented from a representative mouse. (B), Correlation between the percentage of CD11b+ macrophage infiltration in the joints and arthritis disease score. Each symbol represents an individual mouse. (TIF) [file pone.0120327.s001.TIF]

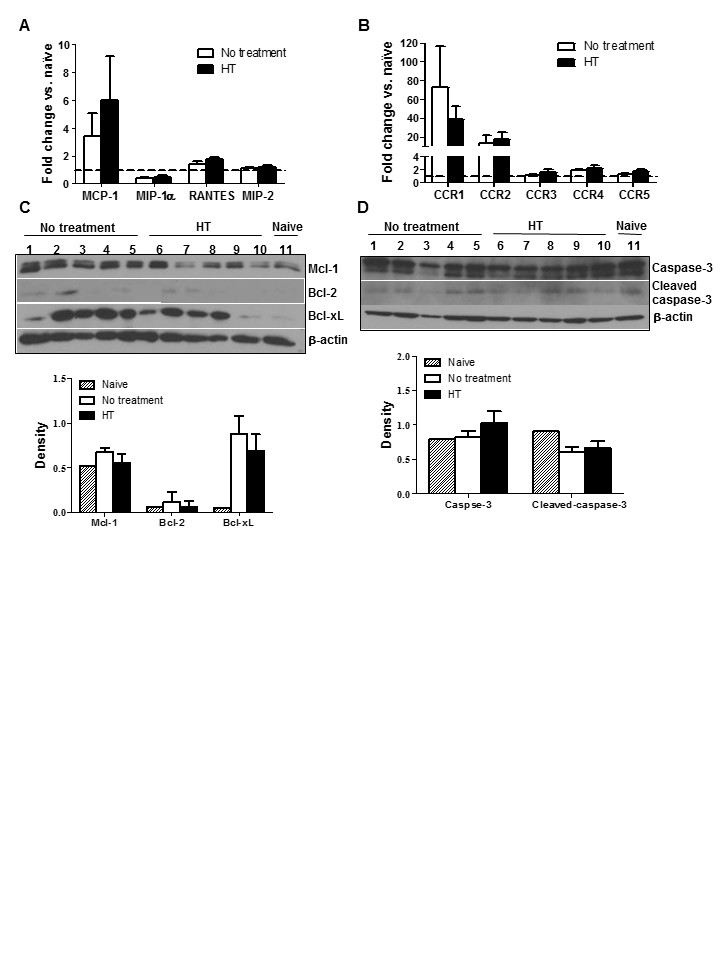

Supplement: S2 Fig — (A-B), RNA was isolated from the joints and mRNA level of chemokines MCP-1, MIP-1α, RANTES, MIP-2 (A) and chemokine receptors CCR1, CCR2, CCR3, CCR4, CCR5 (B) were analyzed by quantitative real-time PCR. The results are presented relative to GAPDH and naïve mice. Baseline expression in naïve mice was shown by the dash line. (C-D), Tissue homogenates were prepared from naïve and CIA mice paws and expression of Mcl-1, Bcl-2, Bcl-xL, caspase-3 and cleaved caspase-3 were detected by Western blotting. Each lane represents different mice. The graph shows the ratio of the band intensity of proteins normalized to β-actin. (TIF) [file pone.0120327.s002.TIF]

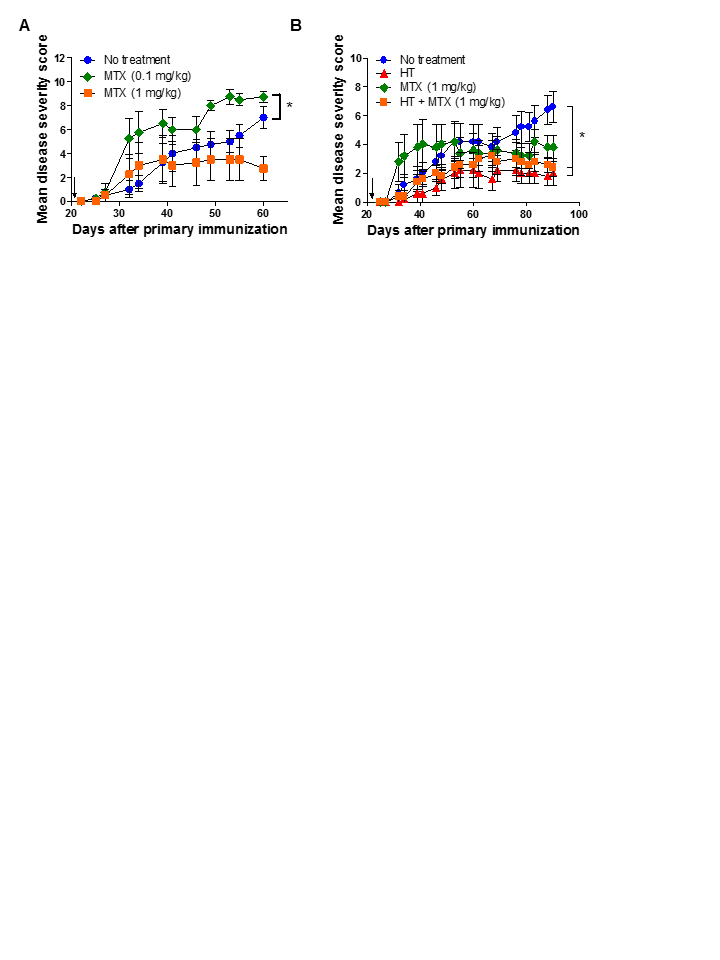

Supplement: S3 Fig — (A to B), DBA1 mice were immunized with bovine CII and received heat treatment (6 hour, 2x/week), MTX (0.1 or 1 mg/kg, 3x/week), heat treatment in combination with MTX or left untreated from day 22. Data are presented as mean disease severity scores ± SEM from 5 individual mice. * p < 0.05, non-parametric Mann-Whitney U-test to compare treated to untreated mice. (TIF) [file pone.0120327.s003.TIF]
